# A taxonomy of early diagnosis research to guide study design and funding prioritisation

**DOI:** 10.1038/s41416-023-02450-4

**Published:** 2023-10-04

**Authors:** Emma Whitfield, Becky White, Spiros Denaxas, Matthew E. Barclay, Cristina Renzi, Georgios Lyratzopoulos

**Affiliations:** 1 ECHO (Epidemiology of Cancer Healthcare & Outcomes), Department of Behavioural Science and Health, Institute of Epidemiology and Health Care, UCL (University College London), 1-19 Torrington Place, London, WC1E 7HB UK; 2 Institute of Health Informatics, UCL, London, UK; 3 British Heart Foundation Data Science Centre, London, UK; 4 Health Data Research UK, London, UK; 5 UCL Hospitals Biomedical Research Centre, London, UK; 6 Faculty of Medicine, University Vita-Salute San Raffaele, Milan, Italy

**Keywords:** Diagnosis, Cancer epidemiology

## Abstract

Researchers and research funders aiming to improve diagnosis seek to identify if, when, where, and how earlier diagnosis is possible. This has led to the propagation of research studies using a wide range of methodologies and data sources to explore diagnostic processes. Many such studies use electronic health record data and focus on cancer diagnosis. Based on this literature, we propose a taxonomy to guide the design and support the synthesis of early diagnosis research, focusing on five key questions:

- Do healthcare use patterns suggest earlier diagnosis could be possible?
- How does the diagnostic process begin?
- How do patients progress from presentation to diagnosis?
- How long does the diagnostic process take?
- Could anything have been done differently to reach the correct diagnosis sooner?

We define families of diagnostic research study designs addressing each of these questions and appraise their unique or complementary contributions and limitations. We identify three further questions on relationships between the families and their relevance for examining patient group inequalities, supported with examples from the cancer literature. Although exemplified through cancer as a disease model, we recognise the framework is also applicable to non-neoplastic disease. The proposed framework can guide future study design and research funding prioritisation.

## Introduction

Researchers and research funders increasingly recognise the imperative to improve diagnosis in medicine [1, 2]. The recent growth in research relating to diagnostic quality and safety is challenging to navigate, due to the different study designs employed to address the same research questions and the lack of consensus on terminology.

We posit that research in the field of diagnostic quality and safety aims to answer five principal questions concerning the diagnostic process:

1. Do healthcare use patterns suggest earlier diagnosis could be possible?
2. How does the diagnostic process begin?
3. How do patients progress from presentation to diagnosis?
4. How long does the diagnostic process take?
5. Could anything have been done differently to reach the correct diagnosis sooner?

A taxonomy (a classification scheme) of different research questions underpinned by theoretical considerations can support a systematic approach to understanding relevant literature and can guide priorities in future research for different conditions. Thus, we discuss study designs and methods best suited to address each of these five questions. Further, we explore how examining variation between and within these study families can advance the understanding of how diagnosis can be improved across patient groups.

## Five principal questions concerning the diagnostic process

### Do healthcare use patterns suggest earlier diagnosis could be possible? (Diagnostic window studies)

The diagnostic window is defined by a pre-diagnostic period where the frequency of healthcare encounters made by an as-yet-undiagnosed cohort (i.e., the group of patients with a pre-specified condition who present because of their underlying condition but who have not as yet received their true diagnosis) increases from ‘background’ healthcare use in the same patients or disease-free controls. The length of the diagnostic window provides a guide to how much earlier it may be possible to diagnose at least some patients with the condition.

Different types of healthcare events can define diagnostic windows, helping to elucidate when the condition becomes detectable in specific ways. Windows defined by primary care consultations provide a generic expression of when as-yet-undiagnosed patients begin to use healthcare differently. Diagnostic windows can additionally be defined by events recorded during the healthcare encounter (e.g., recorded symptoms, prescribed medication, investigations ordered [3, 4]). When conducted in different disease contexts, health systems or eras, diagnostic window studies can reveal differences in diagnostic performance and identify patient groups with the greatest potential for earlier diagnosis [5–7].

The unique strength of diagnostic window studies is that they provide proof-of-concept epidemiological evidence that earlier diagnosis may – in principle – be possible. Empirically demonstrating the existence and length of diagnostic windows is a useful first step in designing diagnostic research. This length not only informs the length of follow-up that should be considered in further studies, but also indicates the period during which quality improvement efforts should focus and the degree of population-level improvement that may be possible.

A principal limitation of diagnostic window studies is that they do not demonstrate the proportion of patients responsible for changes in healthcare use. In theory, a very small number of highly atypical patients could account for detectable changes. Further, diagnostic windows do not provide insight into the exact clinical circumstances of individual patients and do not produce evidence that any specific patient could have been diagnosed any earlier, unlike studies of missed diagnostic opportunities (see below).

### How does the diagnostic process begin? (Prodromal feature studies)

Prodromal features are characteristics that are observed in the as-yet-undiagnosed population at a greater rate than in controls who remain disease-free. Many studies consider prodromal symptoms [8, 9], but there are other possible prodromal features such as abnormal test results [10, 11]. Analyses of large samples of electronic health records have enabled formal identification and quantification of these early signs and symptoms in recent years, alongside their positive predictive values for cancer [8, 12–14].

The main strength of prodromal feature studies is that known prodromal features can be used to guide the diagnostic process, for example in helping to decide whether specialist investigations or referrals are needed. There are several examples of studies estimating the predictive value of symptoms and tests supporting clinical practice guidelines [15–20].

A limitation of these studies is the variability in the length of the period during which features associated with the diagnosis are observed. Some of this variation may be appropriate as different features are likely to be predictive over different periods of time, however much literature in the field of cancer early diagnosis research uses 1- or 2-year periods a priori without justification. Formal evidence from diagnostic window studies can be useful in determining risk periods of appropriate length for studies of early signs and symptoms, particularly for conditions characterised by vague or non-specific symptoms. A new approach uses time-to-event analysis to explore how the association of a feature with diagnosis of a condition changes over time [21].

### How do patients progress from presentation to diagnosis? (Diagnostic pathway studies)

Diagnostic pathways comprise the sequence of different healthcare encounters, investigations, and decisions in a patient’s journey to diagnosis. For example, in symptomatic lung cancer patients, different pathways might encompass visiting a GP with a prodromal feature, being sent for a chest X-ray, referred to a respiratory outpatient department or having an emergency admission [22].

**Fig. 1:**
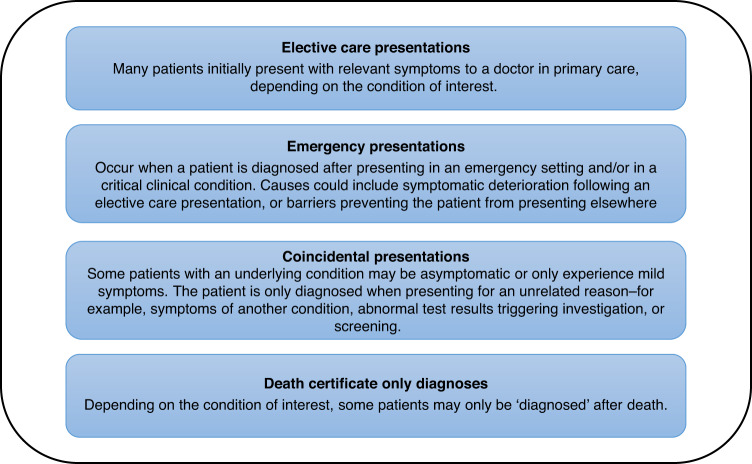
Typical presentations that may be encompassed in the diagnostic pathway. Patients may experience one or more of these presentations during their diagnostic pathway.

A diagnostic pathway begins when a patient first recognises a symptom and ends when the correct diagnosis is made [23, 24]. During this pathway patients typically present to healthcare in three ways, as described in Fig. 1. A small number of additional patients who experience minimal or no prodromal features may have unheralded diagnoses, only recorded on their death certificate [25]. Variation in the proportion of patients presenting through each route may be indicators of our ability to diagnose a condition electively [26, 27].

Walter et al. note two initial patient-dependent stages of the diagnostic pathway – the “appraisal” and “help-seeking” stages [24]. These stages cannot be identified from structured electronic health record data, and require alternative approaches, such as free-text or qualitative interviews with patients and clinicians which may be more susceptible to bias [28–31]. As such, diagnostic pathway studies using EHRs will typically focus on identifying pathways from first presentation through to diagnosis.

Identifying patient-level diagnostic pathways and analysing patterns at population-level produces a map of the routes through which patients typically first present and then progress towards a final diagnosis via tests, prescriptions, and referrals. In the context of diseases with diagnostic guidelines, the proportion of patients diagnosed via guideline-concordant pathways can help assess the success of quality improvement initiatives [32, 33]. Similarly, the “optimality” of different pathways can be assessed by comparing their associations with prognosis and patient experience [34], or through clinician ranking [35].

### How long does the diagnostic process take? (Diagnostic interval studies)

**Fig. 2:**
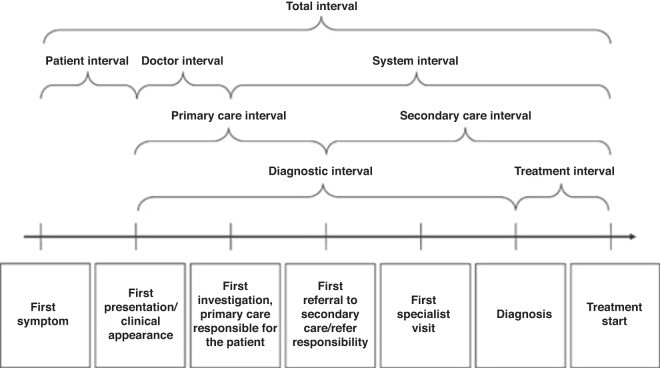
An illustration of the overall milestones and time intervals in the route from first symptom until start of treatment for cancer. Reused from Weller et al. [28], adapted from Olesen et al. [23].

The diagnostic interval for an individual patient is the period between first presentation and diagnosis and is a measure of how long it takes for them to be correctly diagnosed. The ‘total’ interval, from symptom onset to diagnosis or treatment, can be further split into subcomponents, such as the patient interval and the primary care interval [28] (Fig. 2).

Considering diagnostic intervals at population-level allows identification of whether patients with the as-yet-undiagnosed condition are likely to experience diagnostic delay, and quantification of the distribution of any delays. Further, examining changes in average diagnostic intervals can support the evaluation of diagnosis improvement initiatives - such as the introduction of clinical guidelines - and can help to compare performance between and across healthcare systems [36–40]. However, diagnostic intervals should be triangulated with other measures of diagnostic delay to understand whether comorbidities may be artefactually prolonging interval length [7].

The main limitation of both diagnostic pathway and diagnostic interval studies is that, generally, pathways or intervals alone are not sufficient to determine if anything could have been done differently to ensure a specific patient was diagnosed sooner. However, by examining variation with other factors, they can provide an understanding of which patients are at the highest risk of experiencing diagnostic delay and where in the healthcare system delay is most likely to occur. Depending on the disease and healthcare context, these studies may require linkage of multiple datasets to track patient pathways, determine pathway optimality, or measure intervals.

### Could anything have been done differently to reach the correct diagnosis sooner? (Missed diagnostic opportunity studies)

Missed diagnostic opportunities are pre-diagnosis healthcare contacts where post-hoc judgement indicates that alternative decisions or actions could have led to more timely diagnosis [41]. The majority of missed diagnostic opportunities are expected to occur within the diagnostic window and relate to patients with suboptimal diagnostic pathways and prolonged diagnostic intervals. However, there is little empirical research currently demonstrating this.

A current challenge is the unresolved balance between identifying missed diagnostic opportunities both accurately and objectively. One method of identifying missed diagnostic opportunities is manual clinical review [42–44], but this requires resources that limit scalability beyond clinical audit projects.

A second method is to define markers of missed diagnostic opportunities in EHR data. Such phenotypic rules – also termed ‘e-triggers’ - typically incorporate the documented occurrence of an event, combined with a time period during which a subsequent action ought to have followed [45–49]. This allows estimation of the prevalence of a specific missed diagnostic opportunity, but requires prior knowledge of relevant markers.

A third approach is to consider any contacts within the diagnostic window where relevant symptoms have occurred - above those expected coincidentally - as missed diagnostic opportunities [50]. This gives a proxy marker for missed opportunities and still requires manual clinical review to determine whether any individual instance was truly a missed diagnostic opportunity.

Identifying missed diagnostic opportunities can provide both patient- and population- level insight into diagnostic quality and safety incidents that are taking place and their frequency. This could allow for fast and targeted action to improve the diagnostic process. However, any approach incorporating a clinical review component may be subject to hindsight bias [51] – that is, the clinician’s awareness of the patient’s outcome may affect their judgement of whether a missed diagnostic opportunity occurred.

## Discussion

The proposed taxonomy can be used to understand the diagnostic process and systematically organise existing evidence and is summarised in Table 1. We believe there are three additional questions that can be asked within these families of studies to examine why diagnostic quality and safety deviations are occurring and their potential impact.

**Table 1:** Overview of five principal questions of diagnostic quality and safety research.

Question/Study Family	Purpose^a^	Strengths	Limitations	Examples^b^
Do healthcare use patterns suggest earlier diagnosis could be possible?/ Diagnostic windows	Determine if and how much earlier diagnosis may be possible for some patients with the condition. Identify when the condition may first be detectable in different ways. Determine the appropriate length of pre-diagnostic follow-up for other diagnostic quality and safety studies.	• Producing evidence establishing whether earlier diagnosis is possible in patient population(s) of interest • Free of assumptions regarding whether healthcare events relate to the underlying condition • Useful first step when designing diagnostic quality and safety research for conditions	• Can only be evaluated at population level • Does not prove any specific delay happened to any specific patient • Windows differ for different types of healthcare use events	[3, 5, 65]
How does the diagnostic process begin? / Prodromal features	Determine how patients with a condition first present. Identify first clinical signs and symptoms of the condition and, eventually, use them to guide the diagnostic process.	• Critical for supporting clinical guideline recommendations about specialist investigations or referrals • Can be incorporated in decision-support tools (during the consultation)	• Determining when a feature is coincidental, rather than predictive, can be challenging without additional context (e.g., from diagnostic window studies) • Dependent on recording practices of clinicians	[12, 19, 66]
How do patients progress from presentation to diagnosis? / Diagnostic pathways	Map different paths that patients with the underlying condition take through the healthcare system to get a diagnosis. Identify optimal diagnostic pathways by comparing patient outcomes or by clinician ranking.	• Can be used to evaluate how well current diagnostic practice aligns with diagnostic guidelines • Can determine patient groups at greater risk of suboptimal pathways, who might be reached via different targeted improvement efforts • Final linked dataset can provide detailed insights into the diagnostic process	• Linkage of several datasets to track patients through different aspects of healthcare (depending on the healthcare system and condition) requires significant information governance infrastructure, alongside analytical effort, resources, and expertise • Linkage to outcome data or patient survey data needed to determine ‘optimality’ of pathways	[34, 67]
How long does the diagnostic process take? / Diagnostic intervals	Determine how long it currently takes, on average, to diagnose the condition. Identify diagnostic delay at patient- and population- level.	• Useful for evaluating current practice and likely improvements over time • Comparing intervals between and across health systems and patient groups can identify sources of delay and help target interventions	• Challenging to establish time points with certainty (e.g., symptom onset) • On its own, does not establish the reason any specific patient experienced a delay • Confounding by indication may affect the validity of measured intervals, particularly among comorbid patients	[33, 36, 37]
Could anything have been done differently to reach the correct diagnosis sooner? / Missed diagnostic opportunities	Profile direct evidence of specific gaps in diagnostic quality and safety Identify targets for improvement interventions.	• Can be scalable and implementable as part of a ‘learning health system’ • Missed-diagnostic-opportunity-prone scenarios can inform decision support tools	• Accuracy-objectivity balance not yet resolved: accuracy requires human input at the cost of scale, objectivity allows scale at the cost of accuracy	[48, 49, 68]

^a^All families can be used for benchmarking and monitoring of progress in diagnostic quality and safety, though different data infrastructures have facilitated the use of some families (diagnostic pathways, intervals) for benchmarking purposes more than others, at least in the past. All families are suitable for researching inequalities between patient groups.

^b^For each family, we provide selected examples from the cancer literature of some of the most well-known papers.

### What factors are associated with variation in a study family?

For any of the families above we can examine associations with various factors. These include patient factors (age, sex, deprivation, comorbidities, ethnicity, and whether the patient lives alone), healthcare factors (location, type, and size of the healthcare setting), disease factors (cancer morphological type and grade), and era. This can provide insight into mechanisms responsible for prolonged diagnostic delay or convoluted pathways to diagnosis and help target interventions at affected groups.

For example, variations in diagnostic interval with age, and diagnostic pathways with cancer site have been observed [36, 52]. Small differences in diagnostic window length with sex have been shown for primary intracranial tumours [53].

### What relationships exist between these study families?

We may frequently have research questions that relate to multiple study families; for example, are missed diagnostic opportunities more prevalent on certain diagnostic pathways? We can also explore how variation within one family can explain variation within another alongside the factors considered above: if a particular patient group commonly take a suboptimal diagnostic pathway, is this due to the early signs and symptoms they present with, or for other reasons?

For colorectal cancer, for example, women with serious non-gastrointestinal comorbidities who have an emergency presentation have been shown to have a diagnostic window twice as long compared to other patient groups [54, 55]. This shows that for certain patient subgroups targeted improvement efforts could help diagnose patients earlier.

### What impact does variation within a study family have on disease outcomes or patient experience?

We can evaluate the impact of specific diagnostic process experiences by exploring associations between disease outcomes or patient experience and the families above. This helps us to appreciate the consequences of diagnostic quality and safety lapses, and facilitates discussion of which targeted interventions may have the most impact.

Earlier diagnosis of symptomatic cancer is likely to improve survival and quality of life, although benefits vary by cancer site [56]. It has also been shown that patient experience varies with diagnostic pathway for breast, colon, and rectal cancer, with emergency presenters reporting worse and screening-detected patients reporting the greatest satisfaction with care [57].

### Strengths and limitations

The proposed taxonomy explains the main research questions addressed by diagnostic quality and safety research, explores how different study families address these questions and provides a framework against which existing and new research can be organised. Further, it provides an opportunity to standardise terminology used across diagnostic quality and safety research.

A key concern is the extent to which “confounding by indication” may bias research on the diagnostic process. In brief, diagnostic management is influenced by the patient’s health status seen by a clinician [58]. The potential bias this may cause is best illustrated in diagnostic interval studies. Tørring et al. discuss a “U-shaped” relationship between diagnostic interval length and mortality in colorectal cancer patients [59] (also known as the ‘waiting time paradox’ or ‘sicker-quicker’ phenomenon [56, 60–62]). Counterintuitively, there was higher mortality among patients with the shortest diagnostic intervals. This is possibly explained by tumour aggressiveness and stage at presentation, emergency presentations, and multi-morbidity [59]. Concerns have also been raised as to how multi-morbidity may affect the measurement of diagnostic intervals [7]. Researchers should consider methods to assess the presence of confounding, for example comparing intervals by stage or diagnostic pathway groups.

The study families we have described focus on earlier diagnosis as a process measure (achieving diagnosis earlier in time), rather than as a patient/disease outcome (achieving diagnosis at an earlier disease stage). Whilst shorter intervals in the diagnostic process are associated with improved patient outcomes in general [56], Tørring et al. have illustrated that this association varies between patient groups [59]. For cancer, staging classifications are well-developed, but for other conditions disease stage or severity may not be well-defined. Furthermore, we have not considered research concerning overdiagnosis. At present, defining and quantifying overdiagnosis is challenging [63, 64] and generally only possible for patient groups as opposed to individual patients. Nevertheless, consideration of potential overdiagnosis is required when carrying out any of the research we have described.

In developing the taxonomy, we have focused on research using electronic health records. Other possible research methods – such as surveys or qualitative research have not been considered in detail. There may be some overlap in the purposes of the families we describe; for example, both diagnostic windows and intervals could be used to identify conditions where diagnostic delay is a concern. When designing diagnostic quality and safety research, it may be useful to consider the differences between families and how they can address specific questions being asked (Supplementary Table 1).

Finally, the examples we give to support our taxonomy were sourced from the literature on cancer diagnosis, and some of the research we have described may not be possible for certain other health conditions. For example, endometriosis diagnosis and management are entwined and cannot be separated, so defining a date of diagnosis may not always be possible. Without a diagnosis date carrying out diagnostic window studies, for example, would be very challenging.

### Implications

This taxonomy provides a structure against which existing evidence can be compared and organised, helping to elucidate promising targets for further research and improvement efforts. This allows us to borrow methods and adapt findings from diagnostic quality and safety research into other, seemingly unrelated, diseases. This is particularly relevant for conditions where existing evidence may be sparse, such as schizophrenia and rheumatoid arthritis.

The proposed framework can guide research in a sequential fashion; for example, if we want to explore how a specific condition is diagnosed in a specific healthcare system, then we can methodologically work through the families distinguished here (as applicable to the condition) to build the knowledge base, from population- to patient-level.

## Conclusion

We propose a ‘5-question’ taxonomy of diagnostic quality and safety research. The proposed framework can help situate existing research and deepen enquiries into diagnostic quality and safety deviations in conditions such as cancer, where diagnostic delay continues to be prevalent despite growing investment in research. It can also guide rigorous diagnostic quality and safety research in conditions for which existing evidence is sparse. This taxonomy will aid the synthesis of existing evidence, support the design of new studies, and prioritise decisions for research aiming to improve diagnosis in medicine as a whole, and for specific conditions.

### Supplementary information

Supplementary Information
