# Supplementary material for: A taxonomy of early diagnosis research to guide study design and funding prioritisation
Source: Br J Cancer. 2023 Oct 4;129(10):1527–34. doi: 10.1038/s41416-023-02450-4 (PMC10645731; doi:10.1038/s41416-023-02450-4)
Supplement: Supplementary file 1 — Supplementary Information [file 41416_2023_2450_MOESM1_ESM.docx]

# Supplementary information

**Additional notes on study families**

*Diagnostic windows*

We defined the start of a diagnostic window as the time point prior to diagnosis when the frequency of healthcare encounters made by the as-yet-undiagnosed population increases from ‘background’ healthy use. Theoretically, and depending on the healthcare use event being evaluated, the earliest changes in healthcare utilisation could be a decrease relative to the baseline. This would also indicate the start of a diagnostic window, however – to the best of our knowledge – no such windows have been identified as yet.

Various methods for determining diagnostic window presence and length have been proposed^1^. Count models are typically used to model rates of healthcare events of interest, but various approaches have been employed to infer inflection points, including visual inspection and comparison of confidence intervals. Recently a new method has been proposed that overcomes some of the major limitations of other approaches^2^. Variability also exists in the literature regarding the use of case-only or case-control designs to determine background ‘healthy’ healthcare utilisation, and the length of the observation periods within which the diagnostic window is presumed to be contained.

One of the main limitations of diagnostic window studies is that it is challenging to quantify for how many patients earlier diagnosis may be possible. This is particularly true when the healthcare use events being used to define the diagnostic window are not disease specific. However, when specific healthcare events and related features – such as consultations for alarm symptoms or blood test with abnormal findings – are used to define diagnostic windows, they can provide a closer appreciation of mechanisms leading to missed opportunities for prompt assessment^3^. Additionally, the cumulative percentage of patients with specific pre-diagnostic healthcare event(s) of interest can be calculated^4^. On the other hand, these more specific events are also likely to be rarer, and so a larger sample may be required to reliably estimate the diagnostic window.

*Prodromal features*

Although beyond the scope of this taxonomy, prodromal features research can also form the basis for risk prediction, to determine an individual’s risk of being diagnosed with the condition of interest within a time frame^5^. Additionally, we can examine to what extent patients presenting with different features have earlier or more advanced stages of disease, or whether they represent different disease subtypes.

*Diagnostic pathways*

Identifying emergency presentations from electronic health records poses little methodological challenge. However, identifying relevant elective care or coincidental presentations can be more challenging. To do this we first need to know which early signs and symptoms are common and should trigger suspicion of the underlying condition.

It is then possible to work backwards from diagnosis and apply reasonable judgement to determine a ‘first presentation’. However, depending on clinician recording practices and the availability of free-text data, it may be hard to determine whether signs and symptoms that pre-date the diagnosis by a long time were truly caused by the underlying undiagnosed condition or were coincidental (particularly for vague and non-specific symptoms). In these instances, diagnostic window length may aid judgement.

*Diagnostic intervals*

Because of their close ties, diagnostic pathways and intervals are often considered together^6^, reflecting two expressions of the diagnostic journey, the ‘direction’ (quantity and type of steps in the diagnostic process) and ‘time’ (days between each step) respectively. One opportunity for exploration in this area of research concerns a natural extension to this concept – the possibility of measuring a ‘diagnostic velocity’. This would be a quantitative measure of how efficient and well-targeted the diagnostic process was for a patient. It could also reflect the impact long waiting times for referrals, scans, or tests can have on the diagnostic process. To our knowledge, no such ‘diagnostic velocity’ measure has been studied yet.

Supplementary Table 1: Summary of how each study family may help explain each research question

|  | **Diagnostic windows** | **Prodromal features** | **Diagnostic pathways** | **Diagnostic intervals** | **Missed diagnostic opportunities** |
| --- | --- | --- | --- | --- | --- |
| **Do healthcare use patterns suggest earlier diagnosis could be possible?** | The presence of a diagnostic window indicates that earlier diagnosis may be possible for some patients with the condition. The length of the window indicates the extent to which improvement may be possible for those patients. | Identifying prodromal features that occur long before diagnosis can indicate that a condition may be detectable at an earlier stage. | Frequent non-linear pathways can indicate that streamlining may be possible. | Expedited diagnosis might be possible for patient subgroups with long diagnostic intervals on average. | The existence of missed diagnostic opportunities suggests earlier diagnosis could be possible in some patients. |
| **How does the diagnostic process begin?** | The presence of a diagnostic window for a specific symptom or feature implies that this could be an early indicator of the condition^7^. | Working backwards from diagnosis, prodromal features are likely to be the first signs that the as-yet-undiagnosed condition is present. Knowing these features can help identify the first relevant presentations. | To some extent, the healthcare settings in which patients initially present may be driven by the early signs and symptoms they experience. For example, high proportions of emergency presentations may indicate that condition onset is rapid. | Presenting signs and symptoms may explain variation in diagnostic intervals. Long intervals may be associated with symptoms that are uncommon for the as-yet-undiagnosed condition or ‘vague’^8^. | Missed diagnostic opportunities may be more common at the start of the diagnostic process, particularly if patients present with vague or non-specific symptoms. |
| **How do patients progress from presentation to diagnosis?** | The presence of a diagnostic window for a certain kind of healthcare use event may indicate that the event forms part of the diagnostic pathway^4^. | The prodromal features that an individual presents with are likely to influence the investigations that are subsequently carried out^9^. | Diagnostic pathways map the different journeys that patients take from first recognising a symptom to diagnosis, including tests, referrals, alternative diagnoses and prescriptions. | The length of specific sub-intervals can identify whether patients progress from presentation to diagnosis in a timely manner^6^. | Missed diagnostic opportunities may constitute failures to refer patients down specific ‘optimal’ pathways^10^. |
| **How long does the diagnostic process take?** | The length of a diagnostic window for all cause healthcare use can guide the pre-diagnostic follow-up period that should be considered. | No known applications | Identifying different diagnostic pathways and milestones along them is necessary for the measurement of intervals.  Additionally, the number of ‘steps’ in a pathway may function as a proxy for the duration of the diagnostic process. | Diagnostic intervals can be measured for individual patients, and at population level indicate the average time-to-diagnosis. | Missed diagnostic opportunities are indicators of delay in the diagnostic process. The quantity of missed diagnostic opportunities an individual experiences may be a proxy marker of how long the diagnostic process takes. |
| **Could anything have been done differently to reach the diagnosis sooner?** | Patient subgroups with long diagnostic windows may be good targets for diagnostic improvement efforts^11^. | If certain prodromal features are associated with worse outcomes, this may indicate where diagnostic improvements could be targeted^12^. | ‘Suboptimal’ pathways could indicate that improvements may be possible.^13^ | Sub-intervals can indicate where delays in the diagnostic process may be occurring.  If specific patient groups are experiencing delayed intervals, this could indicate that improvements may be possible^14^. | Missed diagnostic opportunities indicate specific healthcare contacts where alternative actions or decisions could have led to more timely diagnosis. |

Boxes highlighted in green indicate the study design typically recommended to address each research question.

# References

1. White, B. *et al.* Does changing healthcare use signal opportunities for earlier detection of cancer? A review of studies using information from electronic patient records. *Cancer Epidemiol* 102072 (2021) doi:10.1016/J.CANEP.2021.102072.

2. Price, S., Wiering, B., Mounce, L. T. A., Hamilton, W. & Abel, G. Examining methodology to identify patterns of consulting in primary care for different groups of patients before a diagnosis of cancer: An exemplar applied to oesophagogastric cancer. *Cancer Epidemiol* **82**, 102310 (2023).

3. Zhou, Y. *et al.* Identifying opportunities for timely diagnosis of bladder and renal cancer via abnormal blood tests: a longitudinal linked data study. *Br J Gen Pract* **72**, e19–e25 (2021).

4. Zhou, Y. *et al.* Imaging activity possibly signalling missed diagnostic opportunities in bladder and kidney cancer: A longitudinal data-linkage study using primary care electronic health records. *Cancer Epidemiol* **66**, (2020).

5. Hippisley-Cox, J. & Coupland, C. Development and validation of risk prediction algorithms to estimate future risk of common cancers in men and women: prospective cohort study. *BMJ Open* **5**, e007825 (2015).

6. Pearson, C. *et al.* Establishing population-based surveillance of diagnostic timeliness using linked cancer registry and administrative data for patients with colorectal and lung cancer. *Cancer Epidemiol* **61**, 111 (2019).

7. Moullet, M. *et al.* Pre-diagnostic clinical features and blood tests in patients with colorectal cancer: a retrospective linked-data study. *The British Journal of General Practice* **72**, e556 (2022).

8. Ozawa, M. *et al.* Symptoms in primary care with time to diagnosis of brain tumours. *Fam Pract* **35**, 551 (2018).

9. Pearson, C., Poirier, V., Fitzgerald, K., Rubin, G. & Hamilton, W. Cross-sectional study using primary care and cancer registration data to investigate patients with cancer presenting with non-specific symptoms. *BMJ Open* **10**, (2020).

10. Singh, H. *et al.* Characteristics and Predictors of Missed Opportunities in Lung Cancer Diagnosis: An Electronic Health Record–Based Study. *Journal of Clinical Oncology* **28**, 3307 (2010).

11. Renzi, C., Lyratzopoulos, G., Hamilton, W., Maringe, C. & Rachet, B. Contrasting effects of comorbidities on emergency colon cancer diagnosis: a longitudinal data-linkage study in England. *BMC Health Serv Res* **19**, (2019).

12. Renzi, C., Lyratzopoulos, G., Hamilton, W. & Rachet, B. Opportunities for reducing emergency diagnoses of colon cancer in women and men: A data‐linkage study on pre‐diagnostic symptomatic presentations and benign diagnoses. *Eur J Cancer Care (Engl)* **28**, (2019).

13. Wiering, B., Lyratzopoulos, G., Hamilton, W., Campbell, J. & Abel, G. Concordance with urgent referral guidelines in patients presenting with any of six ‘alarm’ features of possible cancer: a retrospective cohort study using linked primary care records. *BMJ Qual Saf* bmjqs-2021-013425 (2021) doi:10.1136/BMJQS-2021-013425.

14. Zhou, Y. *et al.* Prolonged Diagnostic Intervals as Marker of Missed Diagnostic Opportunities in Bladder and Kidney Cancer Patients with Alarm Features: A Longitudinal Linked Data Study. *Cancers (Basel)* **13**, 1–12 (2021).
